# Physical Activity-Related Profiles of Female Sixth-Graders Regarding Motivational Psychosocial Variables: A Cluster Analysis Within the CReActivity Project

**DOI:** 10.3389/fpsyg.2020.580563

**Published:** 2020-11-11

**Authors:** Joachim Bachner, David J. Sturm, Xavier García-Massó, Javier Molina-García, Yolanda Demetriou

**Affiliations:** ^1^Department of Sport and Health Sciences, Technical University of Munich, Munich, Germany; ^2^Department of Teaching of Musical, Visual and Corporal Expression, University of Valencia, Valencia, Spain; ^3^AFIPS Research Group, University of Valencia, Valencia, Spain

**Keywords:** self-organizing maps analysis, person-oriented approach, cluster analysis, accelerometer, basic psychological needs, physical activity, physical education

## Abstract

**Introduction:**

Adolescents’ physical activity (PA) behavior can be driven by several psychosocial determinants at the same time. Most analyses use a variable-based approach that examines relations between PA-related determinants and PA behavior on the between-person level. Using this approach, possible coexistences of different psychosocial determinants within one person cannot be examined. Therefore, by applying a person-oriented approach, this study examined (a) which profiles regarding PA-related psychosocial variables typically occur in female sixth-graders, (b) if these profiles deliver a self-consistent picture according to theoretical assumptions, and (c) if the profiles contribute to the explanation of PA.

**Materials and Methods:**

The sample comprised 475 female sixth-graders. Seventeen PA-related variables were assessed: support for autonomy, competence and relatedness in PE as well as their satisfaction in PE and leisure-time; behavioral regulation of exercise (five subscales); self-efficacy and social support from friends and family (two subscales). Moderate-to-vigorous PA was measured using accelerometers. Data were analyzed using the self-organizing maps (SOM) analysis, a cluster analysis including an unsupervised algorithm for non-linear models.

**Results:**

According to the respective level of psychosocial resources, a positive, a medium and a negative cluster were identified. This superordinate cluster solution represented a self-consistent picture that was in line with theoretical assumptions. The three-cluster solution contributed to the explanation of PA behavior, with the positive cluster accumulating an average of 6 min more moderate-to-vigorous PA per day than the medium cluster and 10 min more than the negative cluster. Additionally, SOM detected a subgroup within the positive cluster that benefited from a specific combination of intrinsic and external regulations with regard to PA.

**Discussion:**

The results underline the relevance of the assessed psychosocial determinants of PA behavior in female sixth-graders. The results further indicate that the different psychosocial resources within a given person do not develop independently of one another, which supports the use of a person-oriented approach. In addition, the SOM analysis identified subgroups with specific characteristics, which would have remained undetected using variable-based approaches. Thus, this approach offers the possibility to reduce data complexity without overlooking subgroups with special demands that go beyond the superordinate cluster solution.

## Introduction

Regular physical activity (PA) is an important component of a healthy lifestyle. Engaging in regular PA helps to prevent overweight and obesity and lowers the risk of diseases like diabetes mellitus type II, colon and breast cancers or cardiovascular as well as mental diseases (McKinney et al., 2016; Granger et al., 2017). The World Health Organization (WHO) recommends children and adolescents to engage in at least 60 min of moderate-to-vigorous physical activity (MVPA) per day (World Health Organization [WHO], 2018). Children’s compliance with the WHO guideline is crucial because the participation in regular PA not only leads to positive short- and middle-term effects on their health, but also increases the probability of an active and healthy lifestyle later in adulthood (Hallal et al., 2006; Telama, 2009). However, only a minority of children and adolescents fulfill the minimum guideline of MVPA. Pooled analyses of self-report studies have shown that worldwide 19.7% of children aged 13 to 15 years and 19% of students aged 11 to 17 years reach 60 min of daily MVPA (Hallal et al., 2012; Guthold et al., 2020). Analysis of accelerometer data of over 26,000 5-17-year olds from ten countries indicated an even lower rate with 9% of boys and 1.9% of girls fulfilling the WHO guideline (Cooper et al., 2015). In addition to this gender effect, which is consistently found during adolescence, PA decreases with age (Dumith et al., 2011; Van Hecke et al., 2016). Furthermore, although there have been ambiguous findings, a lower socioeconomic status (SES) of the household tends to have a negative effect on PA levels in children and adolescents (Sallis et al., 2000; Corder et al., 2011; Molina-García et al., 2017; Finger et al., 2018).

By means of an intervention program in physical education (PE), the CReActivity project (Demetriou and Bachner, 2019) aims to promote PA of female sixth-graders attending lower-track secondary schools. The sample was chosen because it represents a specific risk group regarding physical inactivity in terms of age, gender and socioeconomic status (e.g., Van Hecke et al., 2016; Finger et al., 2018). Thirty-three classes and their respective PE teacher participated in the project and were randomly assigned to intervention group (IG) and control group (CG). While the PE teacher behavior remained unaffected in the CG, PE teachers of the IG were provided with 48 fully elaborated lesson plans specifically designed to support students’ basic psychological needs (BPNs) autonomy, competence and relatedness. The lesson plans were divided into the learning domains of football, basketball, gymnastics, health and fitness, swimming and dancing. IG teachers could freely choose which lessons they wanted to implement during the 16-week intervention period. Further details about the intervention program can be found elsewhere (Demetriou and Bachner, 2019).

Self-determination theory (SDT; Ryan and Deci, 2000) and, more specifically, the associated BPN theory (Deci and Ryan, 2000) were chosen as the main foundation for the intervention because they not only allow for the explanation and prediction of human behavior but also include specific mechanisms and instructions on how to actually promote PA behavior via an increase in motivation (Buchan et al., 2012). SDT focuses both on the quantity and on the type of motivation through which human behavior is regulated. According to SDT, six types of motivation can be differentiated and their respective regulation is arranged on a continuum of self-determination (Ryan and Deci, 2000). The state when a person totally lacks motivation is referred to as amotivation. The least autonomous form of motivation is external motivation, when behavior is regulated by external factors such as rewards or imminent penalties. Next on the self-determination continuum is introjected motivation. Introjection describes the circumstance when people are led by external motivation that has already been internalized to some extent. Thus, the behavior does not fully emanate from oneself, but is executed in order to avoid having a bad conscience, to gain pride, or to maintain one’s self-esteem. When a person shows a certain behavior because it is personally valued and considered important, it is referred to as identified motivation. Integrated and intrinsic motivation, finally, are the two most autonomous forms of behavioral regulation. They differ in that intrinsic motivation triggers behavior with the purpose of enjoying the respective activity, whereas integrated motivation means that the person has fully integrated the value of the behavior but still strives for a specific outcome apart from having fun (Ryan and Deci, 2000). SDT proclaims that the satisfaction of the so-called BPN autonomy, competence and relatedness leads to autonomous forms of motivation. Autonomous motivation should in turn promote the respective behavior. A systematic review and meta-analysis (Owen et al., 2014) of cross-sectional studies referring to the relationship of autonomous motivation and PA supports this assumption. Furthermore, several longitudinal studies in the context of physical education (PE) illustrate possible ways to promote PA based on SDT (e.g., Chatzisarantis and Hagger, 2009; Standage et al., 2012; Van den Berghe et al., 2012; McDavid et al., 2014). As a first step, by means of need-supportive teaching behavior in PE classes, the actual satisfaction of BPN can be improved significantly (Standage et al., 2012). Secondly, higher satisfaction of BPN during PE can lead to higher autonomous motivation of students during class (Chatzisarantis and Hagger, 2009; Jaakkola et al., 2012; Standage et al., 2012). Subsequently, autonomous participation in PA during leisure-time (LT) is increased as well, which finally leads to higher PA levels (Chatzisarantis and Hagger, 2009; Standage et al., 2012; McDavid et al., 2014).

To allow for a manipulation check of the CReActivity intervention, the degree of BPN support was assessed. To evaluate the intervention effect, MVPA was measured with accelerometers. To be able to examine possible mediating forces of the intervention effect, BPN satisfaction in PE and leisure-time as well as the quantity and quality of PA-related motivation were assessed. In addition, self-efficacy and social support by family and friends were also measured (Demetriou and Bachner, 2019). As the central construct of the social-cognitive theory (SCT), Bandura defined self-efficacy as “beliefs in one’s capabilities to organize and execute the courses of action required to produce given attainments” (1997, p. 3). Although often deemed redundant to competence, self-efficacy was included as a potential mediator with incremental value because competence and self-efficacy can be distinguished both conceptually and statistically (Rodgers et al., 2014). Several reviews consistently identified self-efficacy as a highly relevant psychological correlate and determinant of PA behavior of children and adolescents (Bauman et al., 2012; Cortis et al., 2017). Further reviews concluded that self-efficacy can help to attenuate the decrease in PA during adolescence and can act as the most powerful mediator in intervention studies trying to promote PA (Lubans et al., 2008; van Stralen et al., 2011). Social support by family and friends was considered as a potential mediator because IG students were supposed to take over a teaching role after school and instruct their friends and families to repeat what they had learned in PE. This way, people around the participants were expected to be affected by the intervention as well, which should finally lead to mutual support. Concrete social support on active lifestyles from parents and peers are independent factors affecting children’s and adolescents’ PA behavior. Several systematic reviews (Mendonça et al., 2014; Laird et al., 2016) indicate the stable and positive association between social support and PA. Specific providers of social support such as family, parents, or friends have been distinguished and their relevance regarding adolescent PA levels has been demonstrated. While peers seem to have an influence during both childhood and adolescence, the effect of parental support on PA tends to decrease as their children reach the age of 16 (Bush and MacDonald, 2015).

Thus, a substantial number of PA-related psychosocial constructs, which in its entirety is also represented in the Youth Physical Activity Promotion Model (Welk, 1999) and other theoretical works (e.g., Laird et al., 2018), was assessed in the CReActivity project. Only with a wide range of psychosocial information available, the idea that PA behavior of an individual is driven by several psychosocial determinants simultaneously could be met and tested. Although this idea had been suggested by the YPAPM (Welk, 1999) and other theoretical models and assumptions (Deci and Ryan, 2000; Vallerand, 2007; Teixeira et al., 2012; Vansteenkiste and Mouratidis, 2016), and had been supported by empirical PA-related studies (Lindwall et al., 2017; Emm-Collison et al., 2020), the majority of studies has applied the traditional variable-based approach (Bergman and Andersson, 2010) that examines relations between different PA-related determinants and PA behavior on the between-person level. Using this approach, the typically occurring coexistence and possible interactions of different psychosocial determinants within one person cannot be examined. Thus, by applying a person-oriented approach to the baseline measures of the CReActivity project, the following research question 1 was examined: Which profiles regarding the assessed PA-related psychosocial variables typically occur in female sixth-graders? If the profiles deliver a self-consistent picture according theoretical assumptions, this would also strengthen the theoretical foundations of the study and would serve as a double-check regarding the validity of the used scales. Therefore, the second research question was posed: Is the coexistence of the psychosocial constructs within the respective profiles in line with theoretical assumptions? Finally, only if the chosen variables lead to profiles that allow for a differentiation between students with different MVPA levels and thus prove to be relevant for PA in this specific sample, the variables can be seen as promising mediators of a subsequent intervention trying to promote MVPA in this sample. Thus, the third research question is: Do the profiles contribute to the explanation of different baseline MVPA levels? To date, this is the first study to examine the relationship between profiles comprising PA-related psychosocial resources and device-based measured MVPA in an adolescent sample. The three research questions led to one central hypothesis. It was assumed that students who have higher MVPA levels, exhibit higher values in BPN support in PE and BPN satisfaction in PE and LT, show a motivation for PA that is rather autonomously regulated, and have higher values in self-efficacy and social support from friends and family.

An appropriate method to examine these questions, given a multitude of variables, is the self-organizing maps (SOM) analysis (Kohonen, 1995). As a cluster analysis, it represents a person-oriented approach, which assumes that various factors used to describe a person, do not develop independently of one another (Bergman and Lundh, 2015). SOM analysis is considered as the predestined method as it provides a number of advantages specifically useful for this study (Herrero-Herrero et al., 2017). First, it includes an unsupervised algorithm for a potentially non-linear data structure. Second, its statistical power is independent from the number of variables included in the analysis. Third, it is a powerful tool to present results in visualized and ostensive form, which quickly illustrates how a complex number of variables are typically associated within a given person. Fourth, by creating superordinate clusters, typical profiles of female adolescents regarding BPN support and satisfaction, motivation, self-efficacy and social support are described and their relevance regarding MVPA can be examined. Fifth, SOM analyses tend to have a higher accuracy in establishing typical profiles compared to more regularly used clustering techniques (Astel et al., 2007; Budayan et al., 2009; Melo Riveros et al., 2019). Lastly, in addition to the superordinate cluster solution, SOM results may instantly hint toward specific subgroups that, to some extent, stand out of their actual cluster and may show special characteristics and needs.

## Materials and Methods

### Participants

The study sample included 475 female sixth-graders. The students were recruited from 20 secondary schools in and nearby Munich. Students and their PE teachers voluntarily decided to participate in the project. Since the sample formed part of an intervention study promoting PA, the requested sample size was calculated under consideration of the intended increase in MVPA. Furthermore, using a formula by Rutterford et al. (2015), the estimated intracluster correlation, the supposed variation in class sizes and the levels of significance and power were considered as well. Sample size calculation resulted in a minimum of 467 students to be included in the analysis.

Students’ mean age was 11.60 years (*SD* = 0.55, *N* = 402). They came from a variety of households with diverse socioeconomic backgrounds. Mean SES was 49.40 (*SD* = 15.98, *N* = 389). SES was assessed by means of four items asking the students to name and describe their parents’ jobs. Answers were rated with regard to the International Socioeconomic Index of occupational status (ISEI) which is again based on the International Standard Classification of Occupation 2008 (ISCO-08) (Ganzeboom, 2010). If the jobs of both parents could be assigned an ISEI value, the job with the higher ISEI was considered (HISEI). A HISEI value could not be determined for every participant because some of the students gave vague answers or did not respond appropriately. The sample was on average normal weight (mean BMI = 19.52, *SD* = 3.73, *N* = 361). The number of indicated BMI values was reduced because some participants, both apparently normal weight and overweight girls, were not willing to be weighed.

The study was approved by the ethics commission of the Technical University of Munich (155/16 S) and the Bavarian State Ministry for Education and Culture.

### Measures

The psychosocial variables were assessed by a paper-pencil questionnaire. The items were translated into German by means of a committee approach. Wording of the items was adapted to the language skills of the sample used in this study to enable students to fully understand the items and to answer properly without the constant help of the assessment team. Prior to the main assessments, the items were pilot tested by means of the pretest procedure (Brislin, 1970; Cha et al., 2007). Results of the pilot study supported reliability and factorial validity of the respective scales.

In this study, the baseline values of the CReActivity project (Demetriou and Bachner, 2019) were used. Data assessments started 5 weeks after the beginning of the school year. When answering the items, students were asked to think about the time period between the beginning of the school year and the assessment day.

#### BPN Support in PE

The 9-item Adolescent Psychological Need Support in Exercise Questionnaire (APNSEQ) was used to measure the support for BPN during PE (Emm-Collison et al., 2016). Participants responded on a 5-point Likert-type scale ranging from 1 (“Strongly disagree”) to 5 (“Strongly agree”). The original items measure support from family, friends, and the PE teacher separately. In a sample comprising adolescents aged 11–15 years, a 3-factor solution of the PE teacher version was supported along with acceptable internal consistencies of the subscales autonomy, competence and relatedness support (Emm-Collison et al., 2016). In the present study, internal consistency of the three subscales was between 0.75 and 0.78.

#### BPN Satisfaction in PE and Leisure-Time

The twelve satisfaction items of the Basic Psychological Need Satisfaction and Frustration Scale (BPNSFS) by Chen et al. (2015) were adapted to measure PA-related satisfaction of BPN in the contexts of PE and leisure-time, respectively. The students had to answer on a 5-point Likert-type scale ranging from 1 (“Strongly disagree”) to 5 (“Strongly agree”). Together with the respective frustration items, a 6-factor model fit the data well in a multinational sample (mean age = 20). Internal consistency of the BPN satisfaction subscales ranged from 0.65 to 0.88 (Chen et al., 2015). Since the items had originally been validated in a sample of mostly young adults, the items were largely simplified in this study. Internal consistency coefficients were between 0.78 and 0.85 (Sturm et al., 2020).

#### Motivation

To assess the quality and quantity of the sixth-graders’ motivation and the respective regulatory styles, the Behavioral Regulation of Exercise Questionnaire 2 (BREQ-2) was used (Markland and Tobin, 2004). The questionnaire comprises 19 items from five subscales, each rated on a 5-point Likert-type scale. The subscale for integrated regulation was not included in the BREQ-2 since it could not be empirically distinguished from identified regulation. In an adult validation sample, a confirmatory factor analysis supported a 5-factor solution with Cronbach’s Alpha being at least 0.73 for the respective scales (Markland and Tobin, 2004). In the present sixth-grade sample, internal consistency lay between 0.65 and 0.82. The subscales exhibited a simplex pattern, meaning that neighboring scales of the self-determination continuum had a higher positive correlation and scales further apart showed stronger negative correlations.

#### Self-Efficacy

The 8-item physical activity self-efficacy scale by Dishman et al. (2010) was used to assess the girls’ self-efficacy regarding PA. The participants responded to a 5-point Likert-type scale ranging from 1 (“Strongly disagree”) to 5 (“Strongly agree”). Originally validated in samples of sixth- and eighth-grade girls, confirmatory factor analyses found good fits for a unidimensional model. Cronbach’s Alpha in the different samples was 0.81 and 0.83, respectively (Dishman et al., 2010). In the present study, Cronbach’s alpha was 0.84 (Bachner et al., 2020).

#### Social Support

The social support scale assessed friend and family support for PA (Dishman et al., 2010). The students had to indicate how often per week they were supported in being physically active by the respective social agents. They could choose from “never,” “once,” “sometimes,” “almost every day,” and “every day.” Using the same samples as for validating the physical activity self-efficacy scale, a 2-factor solution was supported. Cronbach’s Alpha of the two subscales ranged between 0.75 and 0.86 in the different samples (Dishman et al., 2010). In the sample used in this study, internal consistency was 0.74 for friend support and 0.73 for family support.

#### Physical Activity

Leisure-time MVPA was assessed by means of accelerometers (ActiGraph GT3X - wGT3X-BT). Participants had to wear the devices on the right hip for seven consecutive days except during water-based activities. Sampling rate was set to 30 Hz. On weekdays, participants had to put them on at the latest when they started their way to school. They had to wear the accelerometers until 9 pm unless they went to bed earlier. On weekend days, students had to put the devices on as soon as they got up until 9 pm or until they went to bed.

After collecting the accelerometers, the PA data were downloaded. An epoch length of 1 s was used to sum up the raw vector magnitude counts (10-second epochs were used for GT3X models because of their lower memory and battery capacities). It was refrained from using the low frequency extension filter. The algorithm by Choi et al. (2011) was used for wear-time validation. PA data was regarded as valid if a participant had worn the accelerometer for at least 8 h on at least three weekdays and one weekend day. For participants with a positive wear-time validation, the PA data was analyzed with the cut points by Hänggi et al. (2013) to finally estimate the average duration of MVPA per day for each participant. Cut point for MVPA was more than 3360 vector magnitude counts per minute. Hänggi et al. (2013) cut points were selected because they provide an assessment with a high resolution and because they were validated with the same data sampling and processing criteria as the ones chosen for this study (Migueles et al., 2017).

### Procedure

Several weeks before the data assessments started, students and their parents received information letters regarding the purpose and the procedure of the upcoming assessments. Students only participated if they had provided a written consent form signed by them and their parents.

The data assessments were conducted at the beginning of PE classes. The students used codes to guarantee the anonymity of their data. Before the accelerometers were distributed, the students were explained how to put on the accelerometers by themselves. Additionally, the students received an information sheet summarizing the important details concerning the accelerometer use. After all students had put on the accelerometers correctly, they filled out the questionnaire. The actual PE class started as soon as every student had completed the questionnaire. On average, the students took about 20 min to complete the questionnaire.

### Data Analysis

#### Self-Organizing Maps Analysis

The following input variables were included in the SOM analysis:

• BPN support in PE (three variables),
• BPN satisfaction in PE (three variables),
• BPN satisfaction in leisure-time (three variables),
• Amotivation, external regulation, introjected regulation, identified regulation, intrinsic regulation,
• Self-efficacy,
• Social support from friends and family (two variables).

The analysis was conducted with the MATLAB R2018a programme (Mathworks Inc., Natick, MA, United States) and the SOM toolbox (version 2.0 beta) for MATLAB (Vesanto et al., 1999). Questionnaire variables exhibited less than 4% of missing values. The non-significant Little’s (1988) test indicated that they were missing completely at random (χ^2^ = 8,529.19, df = 8634, *p* = 0.787). Missing values were replaced by applying the 5NN imputation method based on the Euclidean distance to the five nearest neighbors of the respective case (Hastie et al., 2001; Troyanskaya et al., 2001).

The SOM analysis classifies the participants and creates typical profiles based on the participants’ similarities regarding their values on the input variables. The SOM procedure comprises three steps (Pellicer-Chenoll et al., 2015): (1) the construction of the neuron network, consisting of 16 × 7 neurons in this study (height × width), (2) the initialization of the SOM, when each neuron is assigned a value or weight for each input variable by two different ways (i.e., randomized and linear initialization), and (3) the training, when the initial values or weights of the neurons are further modified, which is led by two different training algorithms (i.e., sequential and batch) (Oliver Gasch et al., 2016).

The training is an iterative process and the neuronal weights are modified in each iteration in order to find the best solution. This process depends on several factors. Every participant is represented by an input vector. As soon as an input vector is introduced to the neuron network, the neurons try to attract the input vector. A neuron succeeds when it adapts its weight vector until it exhibits the smallest Euclidean distance between its own weight vector and the input vector it tries to gather. Accordingly, every neuron finally has a weight vector most similar to the input vectors it has gathered (Estevan et al., 2019). To what extent a neuron is able to adapt, is again determined by two aspects: (a) the learning ratio, which starts with a high value and decreases during the training process, and (b) the neighborhood function affecting the adaptation magnitude of the winning neuron and the other neurons. More precisely, the winning neuron and its closest neighbors adapt better to a given input vector than the neurons further away in the neuron network. The adaptation is repeated until the training process ends (Pellicer-Chenoll et al., 2015).

Taking into consideration that the final analysis is based on a random procedure (e.g., initialization and entry order of the input vector), the process described above was conducted repeatedly with the number of iterations fixed to 100. In the end, 1600 SOMs were created as two initialization methods, two training algorithms and four different neighborhood functions were computed (i.e., 100 × 2 × 2 × 4). After the quantization and topographical errors had been multiplied, the map with the minimum error was finally chosen (Estevan et al., 2019; Molina-García et al., 2019).

In order to convert the results illustrated by the respective component planes into a more ostensive solution, the neurons were classified into superordinate clusters according to the values on the input variables with help of a k-means method. The number of clusters was restricted to range between 2 and 10 in order to avoid a high number of profiles making the solution difficult to interpret. The final number of clusters depends on the quantization error and the number of participants per cluster. The quantization error describes the average Euclidean difference between the input vectors and the cluster vectors they are allocated to Kohonen et al. (2009). While the quantization error should be low, the clusters should contain a relevant number of participants to truly represent common profiles. By means of these clusters, typical profiles of the sixth-graders with regard to the input variables were described.

Finally, differences between the clusters regarding the respective SOM input variables, BMI, SES, and MVPA were tested by means of univariate ANOVAs (significance level = 0.05) with Bonferroni corrected *post hoc* tests.

## Results

**FIGURE 1:**
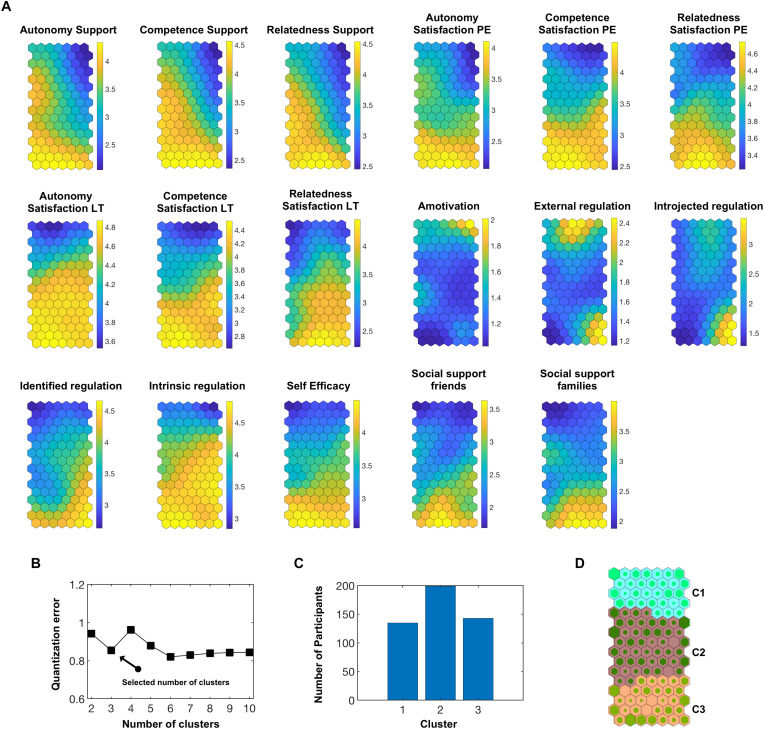
Component planes, clusters and hits obtained by the SOM analysis. **(A)** Component planes of the input variables. Yellow neurons indicate relatively high values, dark blue neurons indicate relatively low values, oriented toward the sample distribution. **(B)** Quantization error according to the possible number of clusters selected. **(C)** Number of participants per cluster for the chosen cluster solution. **(D)** Hits map with the three superordinate clusters C1 – C3. The more a neuron is filled with green, the higher the number of participants assigned to the neuron. PE = physical education; LT = leisure-time.

Figure 1 shows the results of the SOM analysis and how they were derived.

According to the values on the input variables, each participant is placed in a neuron (illustrated as hexagons). Thus, participants who are located in the same neuron exhibit similar values on the input variables. For each input variable a component plane was created (Figure 1A). A given participant is located in the same neuron in each component plane. This illustrates which characteristics regarding the input variables are typically shared by particular groups of the sample.

Making the shared characteristics more visible, SOM and *k*-means analyses indicated that with three clusters, an optimal combination of a low quantization error (Figure 1B) and a considerable number of participants per cluster (≥135; Figure 1C) was found. Therefore, the neurons were classified into three superordinate clusters (Figure 1D).

The descriptive statistics regarding the SOM input variables and MVPA as well as the pairwise comparisons between the three clusters are given in Table 1.

**TABLE 1:** Means, standard deviations, and pairwise comparisons regarding SOM input variables and MVPA.

	Autonomy Support	Competence Support	Relatedness Support	Autonomy Satisfaction PE	Competence Satisfaction PE	Relatedness Satisfaction PE	Autonomy Satisfaction LT	Competence Satisfaction LT	Relatedness Satisfaction LT
Cluster 1	2.83	3.10	3.07	2.62	2.84	3.53	3.81	2.96	2.68
(*n* = 135)	(0.84)	(0.78)	(0.76)	(0.69)	(0.69)	(0.79)	(0.79)	(0.70)	(0.83)
Cluster 2	3.36	3.72	3.67	3.16	3.64	3.98	4.57	3.83	3.46
(*n* = 199)	(0.80)	(0.83)	(0.82)	(0.76)	(0.63)	(0.78)	(0.49)	(0.64)	(0.93)
Cluster 3	4.01	4.32	4.16	3.87	4.33	4.51	4.77	4.39	4.00
(*n* = 141)	(0.69)	(0.60)	(0.60)	(0.63)	(0.54)	(0.51)	(0.38)	(0.58)	(0.84)
Total	3.40	3.72	3.65	3.22	3.62	4.01	4.41	3.75	3.40
(*N* = 475)	(0.90)	(0.88)	(0.87)	(0.85)	(0.84)	(0.80)	(0.68)	(0.84)	(1.01)

	**Amotivation**	**External Regulation**	**Introjected Regulation**	**Identified Regulation**	**Intrinsic Regulation**	**Self-efficacy**	**Social Support Friends**	**Social Support Family**	**MVPA in min/day**

Cluster 1	1.59	1.98	2.08	3.04	3.44	2.83	1.92	2.11	81.58
(*n* = 140)	(0.64)	(0.87)	(0.90)^3, n.s.^	(0.75)	(0.84)	(0.64)	(0.73)	(0.70)	(20.32)^2, n.s.^
Cluster 2	1.19	1.38	1.78	3.66	4.43	3.62	2.51	2.72	85.11
(*n* = 174)	(0.38)^3, n.s.^	(0.49)	(0.68)	(0.72)	(0.55)	(0.63)	(0.80)	(0.75)	(21.17)^1, n.s.^
Cluster 3	1.16	1.67	2.24	4.23	4.74	4.29	3.18	3.56	91.40
(*n* = 163)	(0.42)^2, n.s.^	(0.85)	(1.14)^1, n.s.^	(0.68)	(0.45)	(0.49)	(0.84)	(0.81)	(22.99)
Total	1.30	1.64	2.00	3.65	4.24	3.60	2.54	2.80	85.97
(*N* = 475)	(0.51)	(0.77)	(0.92)	(0.85)	(0.81)	(0.82)	(0.93)	(0.94)	(21.79)

*PE, physical education; LT, leisure-time; MVPA, moderate to vigorous physical activity; n.s., not significant. Data are expressed as means (standard deviations of the means); each pairwise comparison between the clusters was significant (*p* < 0.01) except those marked with superscript numbers indicating non-significant differences to the specified cluster (Bonferroni corrected).*

ANOVAs showed a significant effect of cluster membership on every SOM input variable. Cluster affiliation explained on average 28.4% of the variance in the respective input variables. Cluster 3 represents the positive cluster, as on every BPN support and satisfaction variable as well as on identified and intrinsic regulation, self-efficacy and the two social support variables, students of Cluster 3 exhibited significantly higher values than clusters 1 and 2. Cluster 1, the so-called negative cluster, mainly contained the participants from opposite sides of the sample distribution showing significantly lower values than clusters 2 and 3 in these input variables. Consequently, Cluster 2 constitutes the medium cluster. The negative cluster had on average higher values on amotivation than the medium and positive clusters. The positive cluster comprised both the participants with the lowest and the participants with the highest external regulation. On average, their external regulation was significantly higher than in the medium cluster and lower than in the negative cluster. The participants with the highest values on introjected regulation were allocated to the positive cluster, whose mean value was significantly higher than the one of the medium cluster.

The clusters’ mean MVPA differed significantly (*F*_2,472_ = 7.46, *p* = 0.001, η^2^ = 0.03). With 91.40 min MVPA per day, the positive cluster had significantly higher mean values than the negative cluster (81.58 min) and the medium cluster (85.11 min).

ANOVAs showed no effect of cluster affiliation on SES, but a significant effect on BMI (*F*_2,358_ = 9.66, *p* < 0.001, η^2^ = 0.05) with the participants in the positive cluster having the lowest and the ones in the negative cluster the highest mean BMI.

## Discussion

Based on the assumption that PA-related psychosocial variables do not develop independently from one another within a given person, it was examined which profiles regarding these variables can be observed in female sixth-graders and whether these profiles are in line with the underlying theories (Bergman and Lundh, 2015; Demetriou and Bachner, 2019). By means of a SOM analysis, self-consistent profiles contributing to the explanation of MVPA were differentiated and it is shown that strengthening these resources in female sixth-graders can lead the way to healthy PA behavior.

Referring to research questions 1 and 2, the characteristics of the superordinate clusters are described and their accordance with theoretical assumptions is discussed. With regard to participants in cluster 3, called the positive cluster, it becomes clear that the students who felt most supported in their BPN during PE were also the ones who felt most satisfied in their BPN during PE and LT. This finding is in line with existing literature (Standage et al., 2012; Wang, 2017; Kalajas-Tilga et al., 2019). In line with SDT assumptions (Ryan and Deci, 2000), the PA behavior of these girls was mainly self-determined as they had the highest values on identified and intrinsic regulation (McDavid et al., 2014; Wang, 2017; Kalajas-Tilga et al., 2019). Additionally, the students in the positive cluster exhibited the highest values on self-efficacy and benefited from the highest social support by friends and families, which corresponds to the assumptions of the social-cognitive theory (Bandura, 1997).

A coherent picture evolves when participants of cluster 1, the negative cluster, are added. Since the study sample generally exhibited a healthy MVPA level and had medium to large resources regarding the psychosocial variables, the values of the negative cluster are not worrying when seen from an absolute perspective. However, in relation to the medium and the positive cluster, clear differences emerged. Compared to the rest of the sample, students in the negative cluster reported a relatively low BPN support and BPN satisfaction in PE and LT (Standage et al., 2012; Wang, 2017; Kalajas-Tilga et al., 2019). The girls in the negative cluster showed on average the highest amotivation and their PA behavior was provoked by external regulation to a substantial extent. This finding is again in agreement with the assumptions of SDT (Ryan and Deci, 2000; McDavid et al., 2014; Wang, 2017; Kalajas-Tilga et al., 2019). Furthermore, self-efficacy of this cluster was relatively low and the students received less social support than the rest of the sample, which is again in line with the SCT (Bandura, 1997).

With the exception of external and introjected regulation, the mean values of cluster 2, the medium cluster, were located between the ones of the positive and the negative cluster on every variable. Thereby, the medium cluster completes the generally self-consistent picture, which was revealed by the unsupervised SOM algorithm. Within the participants, the constructs assessed in the CReActivity project (Demetriou and Bachner, 2019) relate to each other in a coherent way, which ultimately leads to a self-consistent superordinate three-cluster solution describing typical profiles of PA-related psychosocial constructs. This indicates that the different psychosocial resources do not develop independently from one another within a given person, which finally supports the use of a person-oriented approach (e.g., Teixeira et al., 2012; Bergman and Lundh, 2015).

The three clusters differed significantly in their average BMI values, with the positive cluster comprising the students with the lowest mean BMI and the negative cluster comprising the ones with the highest mean BMI. More importantly, with reference to research question 3, the students of the positive cluster exhibited a significantly higher MVPA level than the other clusters. A student of the positive cluster exhibits on average around 45 min more MVPA per week than a student of the medium cluster. The mean difference between the positive and negative clusters amounts to almost 70 min per week, which equals one additional day of healthy MVPA behavior each week (World Health Organization [WHO], 2018). Since MVPA was in general on a relatively high level in this sample, the magnitude of the differences between the clusters should be regarded as even more meaningful. Thus, as hypothesized, a self-consistent superordinate cluster solution contributed to the explanation of the students’ MVPA. Thereby, the power of the assessed variables as determinants of PA behavior in children and adolescents is supported and the significance of the SDT for the explanation of PA is underlined (Bauman et al., 2012; Owen et al., 2014; Laird et al., 2016; Wang, 2017; Kalajas-Tilga et al., 2019). This is also in line with recent studies relating profiles of PA-related behavioral regulations with actual PA behavior in adult samples (Lindwall et al., 2017; Emm-Collison et al., 2020). Since MVPA is considered the most important criterion variable here, this result, together with the respective BMI values, further suggests a good criterion validity of the superordinate three-cluster solution (Sarstedt and Mooi, 2019). Equally important, this finding argues for the input variables as promising mediators in a subsequent intervention to promote the MVPA of female sixth-graders (Demetriou and Bachner, 2019).

Thus far, the self-consistent superordinate cluster solution might suggest a strong general factor regarding PA-related psychosocial resources as in most cases the ranking of a given participant was on a highly comparable level across the different psychosocial determinants. However, a main strength of the person-oriented approach (Bergman and Lundh, 2015) in general and the SOM analysis (Kohonen, 1995) in particular becomes clear when looking at the clusters’ values on external regulation. Both the negative and positive cluster included participants with relatively high values on external regulation. By means of the SOM analysis, a non-linear relationship and a dual role of external regulation appeared when looking at the respective MVPA levels. On the one hand, relatively high values in external regulation can constitute the complementary counterpart of a deficit in intrinsic regulation, as can be seen in the negative cluster, which exhibited the lowest MVPA level (Ryan and Deci, 2000). A subgroup of students (*n* = 40) within the superordinate positive cluster, on the other hand, do not meet this conception, as they were both intrinsically motivated (intrinsic regulation ≥4.5) and reported a considerable level of external regulation (≥2.25). In fact, these girls engaged in 95.53 min of MVPA per day, which is higher than the average value of the positive cluster. More than that, they had even higher MVPA values than the students with extremely high values in intrinsic regulation (>4.7) and extremely low values in external regulation (<1.2), who averaged 89.35 min of MVPA per day (*n* = 97). This is similar to the results of a study using self-report measures in a sample of young adolescents, which reported that students with the highest PA levels were the ones who showed high levels of intrinsic regulation and moderate-to-high levels of extrinsic regulation. This supports the assumption that in these cases, external regulation does not harm intrinsic regulation but might even complementarily add to it, which can be beneficial with regard to PA behavior (Harackiewicz, 1979; Ingledew and Markland, 2008). Thus, in these cases, the nature of the external motivation seems to be compatible with the autonomous motivation (Ryan and Deci, 2000; Kilpatrick et al., 2005). Only if the nature of external events comply with personal values and preferences, an originally external motivation can be introjected and finally transformed into more autonomous forms of regulation (Teixeira et al., 2012). This requirement can be met when external events or rewards are conducive to the BPN (Vansteenkiste et al., 2010). An example would be positive feedback, which is deemed to support competence. The fact that students in the subgroup with high levels in intrinsic regulation and moderate levels in external regulation are the only ones with moderate-to-high values in introjected regulation, indicates that these requirements were sufficiently met in these students. Additionally, this shows that MVPA of some adolescents can be regulated by external, introjected and autonomous forces at the same time, which is in line with the findings of other studies that examined groups of adults and older adolescents, respectively (Lindwall et al., 2017; Bechter et al., 2018). Furthermore, this result suggests that prevention or intervention programs should not only focus on supporting autonomous motivation, because the promotion of external motivation does not necessarily undermine the existing autonomous motivation, but can support or add to it (e.g., Ingledew and Markland, 2008). Moreover, this indicates the need and the potential of tailored interventions designed to promote PA of distinct subgroups with specific baseline characteristics and conditions (Hagger, 2010; Bock et al., 2014; Gut et al., 2020). For example, MVPA of the subgroup with the highest values in intrinsic regulation and the lowest values in external regulation might be enhanced in an intervention using the potential of external incentives that are compatible with their existing self-determined motivation (Ryan and Deci, 2000; Kilpatrick et al., 2005; Vansteenkiste et al., 2010).

Using a variable-based approach with analyses conducted on an aggregated group level (Bergman and Lundh, 2015), the findings on subgroups with different characteristics regarding external regulation would not have appeared. Instead, the magnitude of the correlation between external regulation and MVPA would have decreased. In the person-oriented SOM analysis, however, one benefits from obtaining results both in numerical form and results that are presented graphically (Figure 1A). This way, after the reduction of the data into an ostensive superordinate cluster solution, the visual presentation of the results makes it possible to quickly identify potential subgroups within the clusters that might require specific attention. The advantage of the visual presentation of SOM results is not given in other clustering techniques like k-means. Together with the advantages outlined above, these points underline the suitability of SOM for accurately reducing data complexity without the risk of overlooking subgroups with special demands that go beyond the superordinate cluster solution. Thus, the SOM analysis proves valuable in explaining PA behavior, as it is able to go beyond the examination of associations between variables. Rather, it enables the differentiation between distinct groups of people whose respective demands and deficits can then be answered more specifically by developing tailored interventions (Lindwall et al., 2017; Emm-Collison et al., 2020; Gut et al., 2020).

However, some limitations of this study need to be addressed. On the one hand, the main goal of a cluster analysis is to reduce the complexity of the given information transforming it into an applicable solution (Hofstetter et al., 2014). On the other hand, reducing the information usually leads to a decreased precision (Steinley, 2006). The ultimate task is to allocate the participants precisely, without increasing the number of clusters to an extent that ultimately counteracts the original purpose of a cluster analysis. The low quantization error of the superordinate three-cluster solution found here indicates a good trade-off, which is further underlined by the fact that the error would not have been substantially lower with a higher number of clusters. However, since the superordinate three-cluster solution is based on a combination of all 17 input variables, it does not differentiate equally well for each input variable between participants with relatively high, medium and low values. This can be seen in the medium cluster, which showed a less homogeneous profile. Some students, for example, reported a high competence support in PE, but actually perceived themselves as less competent than other students reporting the same level of competence support. Although this might motivate further research into what might interfere with the path from competence support to competence satisfaction among these students, this can be seen as a minor deficit in selectivity for the benefit of a parsimonious and applicable cluster solution.

In addition, according to the Health Action Process Approach (HAPA; Schwarzer, 2008), the process of adopting a healthy behavior comprises both motivational components resulting in an intention, and volitional processes that finally lead to the actual behavior. The psychosocial determinants used to explain PA behavior in this study solely focused on constructs of the motivational spectrum. Volitional constructs could help to bridge this intention-behavior gap to some extent as they are more proximal to the actual behavior. By means of a person-oriented approach in a sample of ninth-grade students (Gut et al., 2020), levels in motivational and volitional psychosocial constructs were examined. For most adolescents, motivation and volition were highly similar. However, there was a small group with high self-determined motivation and strong intention but low values in action planning whose exercise and sport activity level was clearly lower compared to the participants with a high motivation and high volition profile. This suggests that the explanation of PA behavior could have been greater by including volitional constructs as well.

Furthermore, the findings should be verified in a more diverse sample with regard to gender and age. In addition, it would be interesting to replicate the analysis in a sample with lower MVPA levels.

## Conclusion

Based on the theoretical framework of the YPAPM, the SOM analysis revealed three superordinate clusters of female sixth-graders with relatively high, medium and low psychosocial resources regarding PA. The three-cluster solution represents a self-consistent picture in accordance with the theoretical assumptions and contributes to the explanation of MVPA. The results support the use of a person-oriented approach, as the SOM analysis could show that the different psychosocial resources do not develop independently of one another within a given person (e.g., Teixeira et al., 2012; Bergman and Lundh, 2015). In addition to the superordinate cluster solution, the analysis enabled the detection of a specific subgroup, which provided insights into the coexistence and interaction of behavioral regulations in PA. The psychosocial variables assessed in the CReActivity project are deemed promising mediators in promoting MVPA in this sample. Finally, the SOM analysis proved to be a valuable tool for differentiating between distinct profiles regarding PA-related psychosocial resources and for identifying specific subgroups with special characteristics, which would have remained undiscovered using variable-based approaches.

## Data Availability Statement

The original contributions presented in the study are included in the article/Supplementary Material, further inquiries can be directed to the corresponding author.

## Ethics Statement

The studies involving human participants were reviewed and approved by Ethics commission of the Technical University of Munich. Written informed consent to participate in this study was provided by the participants’ legal guardian/next of kin.

## Author Contributions

YD, JB and DS designed the study. YD and JB engaged in the funding acquisition. JB and DS conducted the data collection. XG-M and JB analyzed the data. XG-M, JM-G, and JB interpreted the results. JB wrote the original draft of the manuscript. YD and JM-G supervised the cooperation. All authors contributed to the revision of the manuscript and have read and approved the submitted version.

## Conflict of Interest

The authors declare that the research was conducted in the absence of any commercial or financial relationships that could be construed as a potential conflict of interest.
