# Supplementary material for: Physical Activity-Related Profiles of Female Sixth-Graders Regarding Motivational Psychosocial Variables: A Cluster Analysis Within the CReActivity Project
Source: Front Psychol. 2020 Nov 11;11:580563. doi: 10.3389/fpsyg.2020.580563 (PMC7686241; doi:10.3389/fpsyg.2020.580563)
Supplement: Supplementary file 1 [file Presentation_1.PDF]

```matlab
% Script for the analysis of Physical Activity Motivation using
% Self-Organizing MAPs
% This function only works in Matlab if SOM toolbox is installed.

clear;close;clc;
%Change for the correct path
cd('/Users/xagarmas/MEGA/Joaquim/Estudio Experimental Septiembre 2019');
%Change the name of the data file
 [data,txt,raw]=xlsread('data3.xlsx');

sData= som_data_struct(data,'name','JOAQUIM_prenuevo','comp_names',...
{'AutonomySup','CompetenceSup', 'RelatednessSup',
'AutonomySatPE','CompetenceSatPE','RelatednessSatPE'...

'AutonomySatLT','CompetenceSatLT','RelatednessSatLT','Amotiv','Ext','Intr
o','Iden','Intri'...
     'SelfE','SocialSuppFrien','SocialSuppFami'});

cd('/Users/xagarmas/MEGA/Joaquim/Estudio Experimental Septiembre
2019/SOMresults');

%% Data Normalization
sData = som_normalize(sData,'range');

save('SOM_JOAQUIM');

%% Building SOM%%

S=1;
for i=1:2;
    if i==1;
        training='seq';
    else
        training='batch';
    end

    for j=1:2;
        if j==1;
            inicialization='randinit';
        else
            inicialization='lininit';
        end
        for k=1:4;
            if k==1;
                neig='gaussian';
            elseif k==2;
                neig='cutgauss';
            elseif k==3;
                neig='ep';
            else
                neig='bubble';
            end
            for l=1:100

sM=som_make(sData,'init',inicialization,'algorithm',training,'neigh',neig
);

                % SOM QUALITY %
                [qe(S),te(S)] = som_quality(sM,sData);
```

```
                namesom=['som_' num2str(S)];
%name of the SOM
                feval('save', namesom, 'sM');
%save SOM
                S=S+1;
            end
        end
    end
end
save ('qe','qe');
save ('te','te');

% Select the best SOM

cd('/Users/xagarmas/MEGA/Joaquim/Estudio Experimental Septiembre
2019/SOMresults');
quality=qe.*te
[quality pos]=sort(quality);
load(strcat('som_',num2str(pos(1))));

% To make clusters
clusters=10;
[color,b,kmeans]=som_kmeanscolor(sM,clusters,'hsv')
```
