# Supplementary material for: Physical Activity-Related Profiles of Female Sixth-Graders Regarding Motivational Psychosocial Variables: A Cluster Analysis Within the CReActivity Project
Source: Front Psychol. 2020 Nov 11;11:580563. doi: 10.3389/fpsyg.2020.580563 (PMC7686241; doi:10.3389/fpsyg.2020.580563)
Supplement: Supplementary file 2 [file Presentation_2.PDF]

Please follow the instructions to use the .m file MATLAB code:

1. Install the SOM toolbox of the Laboratory of Computer and Information Science from the Helsinki University of Technology (http://www.cis.hut.fi/)
2. In line 7 change the path of the data file.
3. In line 9 change the name of the data file.
4. In lines 11-17 change the names of the SOM and the names of the components.
5. In line 19 change the path in which you want to save the SOM files.
6. In line 25 change the name of the file with the data that will be used to construct SOM.
7. Lines 27-71: SOM are obtained. In total 1600 SOMs are calculated using different parameters (i.e., initialization methods and training algorithms and neighborhood functions).
8. In line 75 change the path to the folder with the saved SOM files (the same as point 5).
9. Lines 77-79: the best SOM based on the lowest product of quantization and topographical errors is selected.
10. Lines 82-83: k-means clusters are obtained.
